# Monitoring Ca19‐9 during pancreatic cyst surveillance: Better safe than sorry?

**DOI:** 10.1002/ueg2.12449

**Published:** 2023-08-13

**Authors:** Giampaolo Perri, Santhi Swaroop Vege, Giovanni Marchegiani

**Affiliations:** ^1^ Department of General and Pancreatic Surgery Verona University Hospital Verona Italy; ^2^ Division of Gastroenterology and Hepatology Department of Internal Medicine Mayo Clinic Rochester New York USA; ^3^ Department of Surgical Oncological and Gastroenterological Sciences (DiSCOG) University of Padua Padua Italy

The present issue of *United European Gastroenterology Journal* features a paper by Iris JM Levink et al., assessing “the additive value of CA19‐9 monitoring in a pancreatic cyst surveillance program” using the data generated by the PACYFIC‐registry work group. Serum carbohydrate antigen 19‐9 (CA 19‐9) is a cell surface glycoprotein complex with a role in cell‐to‐cell recognition processes and needs for expression of Lewis blood group antigen (absent in 5%–10% of population). It is the most used tumor marker for the diagnosis, prognosis, and management of pancreatic cancer.^1^ Consequently, it is also used as a marker of possible malignant transformation in pancreatic cysts (PC). International^2^ and European^3^ Guidelines consider an elevated (>37 U/mL) serum CA19‐9 as a “worrisome feature” and a “relative indication for surgery”, suggesting an implicit higher risk of malignancy, despite not being enough to represent a standalone absolute indication for resection. American college of gastroenterology guidelines included elevated CA 19‐9 as a high‐risk characteristic for PC and an indication for endoscopic ultrasound and fine‐needle aspiration cytology.^4^ A recent meta‐analysis reported a pooled sensitivity of 40%, a specificity of 89%, and an OR of 4.34 with levels >37U/mL.^5^

Some intrinsic features may limit the use of CA19‐9 in clinical practice, including not being produced by 6% and 22% of the Caucasian and non‐Caucasian populations, respectively, and being elevated in other conditions, such as cholestasis, cirrhosis/hepatitis, diabetes, hypothyroidism, and many other types of cancer.^1^ However, the authors raised a specific concern regarding CA19‐9's reliability as an early marker of progression to high grade dysplasia (HGD) or invasive cancer (IC) in PCs. Their effort should be commended as their study represents a prospective evaluation of patients undergoing surveillance, while current recommendations regarding CA19‐9 are based mostly on retrospective studies. They found that an “abnormal” CA19‐9 according to the currently accepted cut‐off (>37 U/mL) does not predict malignant transformation (including HGD) during surveillance. Moreover, its monitoring may, if anything, cause substantial harm by shortening surveillance intervals and performance of unnecessary surgeries (60% of patients undergoing surgery with an elevated value had benign disease) due to the high rate of false positive results. Notably, half of the patients with HGD/IC had a non‐elevated Ca19‐9. Interestingly, a large single‐institution cohort recently showed similar results, with 15% of patients with low grade IPMNs having an elevated CA 19‐9 and, conversely, 72% of patients with HGD/IC having a normal tumor marker, despite the presence of an obvious selection bias (surgical series of pathologically proven IPMNs).^6^

Indeed, the problem of CA19‐9 in this setting seems to be two‐fold. Biologically, it probably represents a marker for advanced disease and therefore less suitable for early detection (low sensitivity). At the same time, the currently accepted cut‐off of 37 U/mL seems prone to unacceptably low specificity in patients surveilled for PCs. In their paper, the authors also propose a new cut‐off (≥133U/mL), increasing the specificity of the test to 99%. However, as a result, only 9/685 (1%) patients would be affected by an abnormal CA19‐9 as a marker of progression in the decision making.

In a broader sense, whether to detect early malignancy in surveilled PC, to unveil micro‐metastatic disease in newly diagnosed pancreatic cancer, or to assess the response to chemotherapy during re‐staging, the challenges in the field of CA19‐9 interpretation seem to be similar again. Probably, there is a finite amount of knowledge to be extracted from this marker, regardless of the different cut‐offs, curves, or nomograms adopted.^7^ Despite the fact that decision making cannot be based solely on its elevated value, CA19‐9 is still currently useful, especially when viewed in the context of the entire spectrum of each patient's characteristics. New International Guidelines on PC are in the pipeline, and CA19‐9 will likely maintain its not negligible role. However, advances in this field appear to be more in the form of new biomarkers like next generation sequencing for specific mutations,^8^ serum proteome,^9^ tumor microenvironment and microbiome,^10^ and deep learning and artificial intelligence.^11^

## CONFLICT OF INTEREST STATEMENT

The authors declare no conflicts of interest.

## Data Availability

Data sharing is not applicable to this article as no new data were created or analyzed in this study.
